# Inferring gene regression networks with model trees

**DOI:** 10.1186/1471-2105-11-517

**Published:** 2010-10-15

**Authors:** Isabel A Nepomuceno-Chamorro, Jesus S Aguilar-Ruiz, Jose C Riquelme

**Affiliations:** 1 Dpt Lenguajes y Sistemas Informaticos, Universidad de Sevilla, Seville, Spain; 2 School of Engineering, Pablo de Olavide University, Seville, Spain

## Abstract

**Background:**

Novel strategies are required in order to handle the huge amount of data produced by microarray technologies. To infer gene regulatory networks, the first step is to find direct regulatory relationships between genes building the so-called gene co-expression networks. They are typically generated using correlation statistics as pairwise similarity measures. Correlation-based methods are very useful in order to determine whether two genes have a strong global similarity but do not detect local similarities.

**Results:**

We propose model trees as a method to identify gene interaction networks. While correlation-based methods analyze each pair of genes, in our approach we generate a single regression tree for each gene from the remaining genes. Finally, a graph from all the relationships among output and input genes is built taking into account whether the pair of genes is statistically significant. For this reason we apply a statistical procedure to control the false discovery rate. The performance of our approach, named REGNET, is experimentally tested on two well-known data sets: *Saccharomyces Cerevisiae *and E.coli data set. First, the biological coherence of the results are tested. Second the E.coli transcriptional network (in the Regulon database) is used as control to compare the results to that of a correlation-based method. This experiment shows that REGNET performs more accurately at detecting true gene associations than the Pearson and Spearman zeroth and first-order correlation-based methods.

**Conclusions:**

REGNET generates gene association networks from gene expression data, and differs from correlation-based methods in that the relationship between one gene and others is calculated simultaneously. Model trees are very useful techniques to estimate the numerical values for the target genes by linear regression functions. They are very often more precise than linear regression models because they can add just different linear regressions to separate areas of the search space favoring to infer localized similarities over a more global similarity. Furthermore, experimental results show the good performance of REGNET.

## Background

In the area of microarray data analysis, inferring gene-gene interactions involved in biological function is a relevant task. Over the past few years several statistical and machine learning techniques have been proposed to carry out the inferring task of gene-gene interactions or gene regulatory networks. Clustering algorithm represents one of the first approaches to support the identification of regulatory modules [1,2]. These approaches are motivated by a simple idea which is still widely used in functional genomic. It is called the guilt-by-association heuristic: co-expression means co-regulation, i.e. if two genes show similar expression profiles, they are supposed to follow the same regulatory regime.

In order to formalize the idea of similar expression, several statistical measures have been proposed as solution. In correlation methods, interactions are inferred using correlation statistics as pairwise similarity measures between gene expression profiles over multiple conditions, as for example in [3]. In this kind of methods, if the correlation between gene pairs is higher than a threshold value, then it is considered that these gene pairs interact directly in a signaling pathway and are relevant in a biological way [4-6]. These methods build gene co-expression networks, also known as gene association, gene interaction or gene relevance networks. These networks provide a framework for assigning biological function to group of genes as it was argued in [7]. Correlation coefficient is widely used as a way of obtaining an association measure between two random variables but does not provide a causal measure between them. However, correlation is still informative about the underlying structure [8]. The causal properties that can be inferred from correlations have been investigated in [9,10].

Correlation-based methods are very useful to determine whether two genes have a strong global similarity over all conditions from the data set. This is an important constrain as there might exist a strong local similarity over a subset of conditions, which could not be detected with global similarity measures. In addition, many pairs of genes show similar behavior in gene expression profiles by chance even though they are not biologically related [11], i.e. the significance of the results should be assessed in interaction networks.

On the other hand, Gaussian graphical models (GGM) are a full conditional independence model. These models try to explain the correlation between two genes by the rest of the genes and they are a popular tool to represent gene association network [8,12,13]. Recently, [14] has proposed estimating partial correlations to attach lengths to the edges of the GGM, where the length of an edge is inversely related to the partial correlation between a gene pair. As a drawback, these models are hard to estimate if the number of samples is small compared to the number of variables. In contrast to GGMs, other models try to explain the correlation between two genes not by the rest of the genes, but only by single third genes. This idea can also be implemented using sparse Gaussian graphical model based on partial correlation [15] or conditional mutual information to test for first-order independence [16-18].

Bayesian networks try to explain the dependence between genes if there are no subset of other genes that explain the dependency [19]. An example of Bayesian networks can be found in [20] where a *stochastic expectation and maximization *algorithm is used to learn a probabilistic model, and regression trees are used to learn graph topologies that maximize Bayesian scores. Recently, [21] has revised the approach before using an ensemble method, and [22] has incorporated prior knowledge from literature on Bayesian networks. Also, several approaches have been developed to build Boolean networks [23], or to infer regulatory rules [24,25] using machine learning principles.

In this paper, we present a novel method inspired by model trees as a way to detect linear dependencies between genes and to set a group of gene-gene dependencies. From that set, our method provides as gene-gene interactions all those significant dependencies in a statistical sense. Then, it builds undirected dependency graphs (UDGs) from these gene-gene interactions. Furthermore, our method analyzes which dependencies between genes are considered as a discovery by means of the Benjamini and Yekutieli procedure [26]. This statistical procedure enables the control of the expected proportion of false discoveries among all the discoveries made. One of the main contributions of our approach is that it addresses the issue of searching for local similarities arising from conditional regulatory relationships -instead of global similarities.

The remainder of this paper is organized as follows. In Section *Method*, a detailed explanation of the methodology and the algorithm are presented. In Section *Results and Discussion*, experimental results tested on an in silico benchmark suite of datasets, yeast and E.coli data are provided. Finally, Section *Conclusions *summarizes the most relevant conclusions and future research directions.

## Method

Correlation methods are focused on the global match of two gene expression profiles, analyzing each possible pair of genes. Instead, our approach analyzes each gene in an iterative way. At each iteration a gene is taken as target gene and the remaining genes as input for splitting the search space. In each subspace generated by that division, a linear model is built to identify a linear dependency between the target gene and a subgroup of genes, i.e. the target gene expression values are estimated by this subgroup of genes involved in that linear model. As a consequence, the dependency between the genes is not calculated for the complete gene expression profile, but for a localized subspace of the profiles using M5' model tree algorithm.

**Figure 1:**
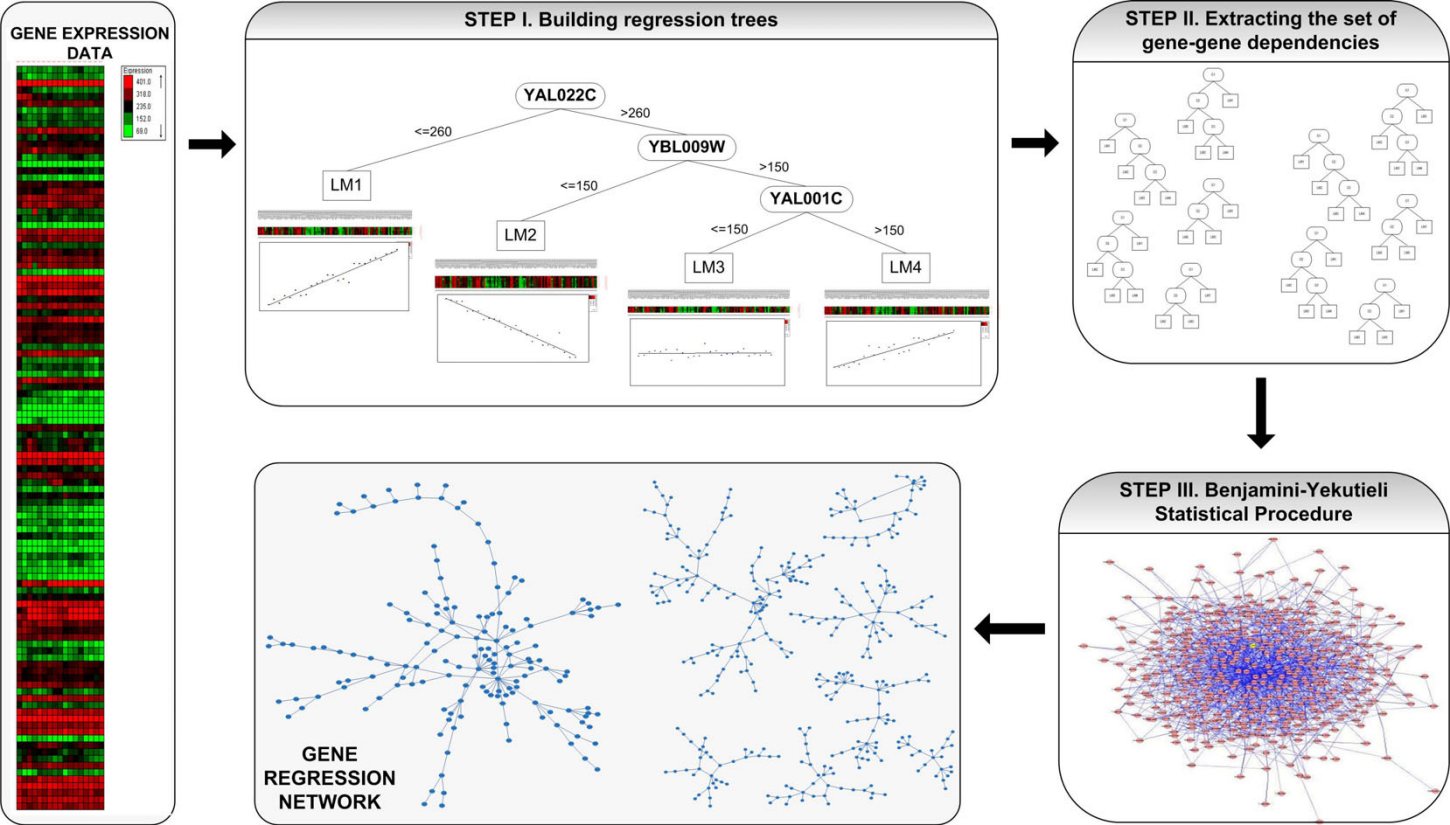
**Schematic view of the proposed method**. In the first step, for each gene (target gene) a model tree is generated, which provides a partition of the space. Linear regression functions are built in the leaves of the tree. These linear regression functions can be seen as prototypes to estimate the value of the target gene. Genes involved in the linear regression functions might be identified as potential dependencies. This is an iterative process that is made for each gene taking the remaining genes as input to build the model tree, and it provides a set of hypothetical gene-gene interactions. Only the model trees with low prediction error will be conserved. Next, the Benjamini-Yekutieli statistical procedure is applied in order to assess the significance of the dependencies.

Our method consists of three steps as it is depicted in Figure 1. The first step involves building M5' trees. M5' is a model tree algorithm, an extension of regression tree algorithms [27], which has several linear models, each one of them built in a leaf of the tree. The aim of this step is to obtain a set of genes associated to other genes from their prediction ability by means of linear regression functions. We use model trees because these representations work like several linear regression functions at the same time, each of them identified by a leaf in the tree. The main advantage of this methodology is that each regression is specialized in a specific area of the search space, i.e. in a local subspace of gene expression profiles, hence the model tree is generally more accurate than a global linear regression.

The second step implies the extraction of the set of gene-gene dependencies from the forest of trees obtained by the previous step. Specifically, our approach considers which hypothetical evidences of gene-gene dependency exist between the target gene and every gene participating in the linear regression functions of the target gene.

Finally, the third step involves learning a graph model of gene co-expression network by assessing the significance of the set of hypothetical evidences. Many sets of genes show similar behavior in expression profile by chance even though they do not share the same biological function. Therefore, the aim of this step is to minimize the number of false discoveries among all those discoveries made in the previous step. For that reason, we apply a statistical procedure to control the false discovery rate instead of the increase of type I error when a family of hypotheses is being tested simultaneously. The reliability of our method is strengthened by applying the Benjamini-Yekutieli statistical procedure to assess the significance of the results.

### Building model trees

The first work on regression trees dates from [28], although the most popular reference is the seminal work of [29]. Later on, [30] introduced the system M5. It builds multivariate trees using linear regression functions at the leaves. M5' is introduced in [31], a rational reconstruction of Quinlan's M5 algorithm. Throughout the description of model tree, we will refer to gene as attribute, and sample as instance space.

**Figure 2:**
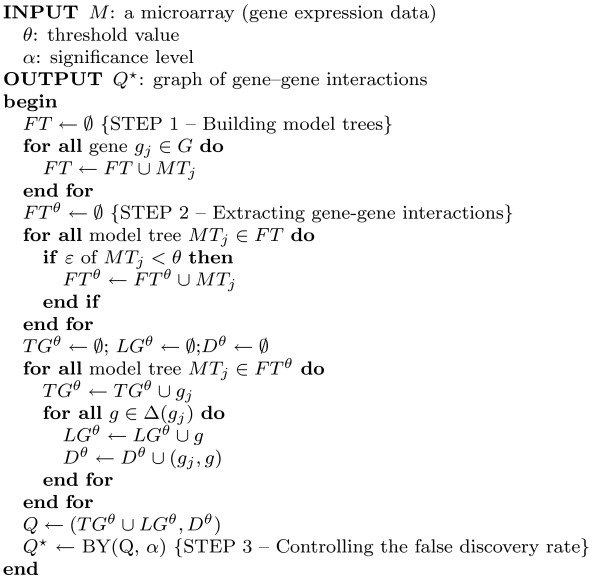
**Pseudocode**. Pseudocode of REGNET.

The algorithm M5' is divided into two phases. First, a tree is built by a decision-tree induction algorithm, and second, a pruning procedure is applied. Given a gene as a target, M5' constructs a tree by recursively splitting the instance space. In this decision-tree induction algorithm the splitting criterion is based on treating the standard deviation, i.e. the attribute which maximizes the expected error reduction is chosen. After the tree has been built, a linear regression function is obtained for every internal node of the tree and the regression models are reduced by dropping attributes to minimize the estimated error on future data. The number of attributes in the linear regression functions decreases and the average error will offset over the training example. After this has been done, every subtree is considered for pruning. Pruning takes place if the estimated error for the linear regression function at the root of a subtree is smaller than or equal to the expected error for this subtree. After pruning is done, M5' applies a smoothing process to compensate sharp discontinuities that occur between adjacent regression models at the leaves of the tree. Finally, M5' has an associated relative error *ε *that will be used to reject some of the trees, those with low precision. The result is a forest of trees (*FT*^*θ *^in Figure 2). This algorithm is described in [30,31].

**Figure 3:**
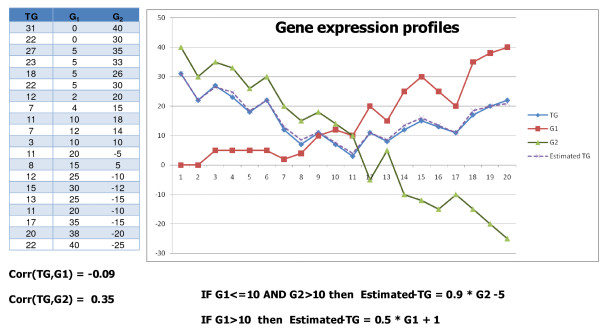
**Hypothetical example of localized similarities**. The table represents the gene expression values from 20 samples. The correlation coefficients between the target gene *TG *and the two other genes are weak (*ρ*(*TG, G*_1_) = -0.09 and *ρ*(*TG, G*_2_) = 0.35). However we can observe in this hypothetical example two strong localized similarities detected by construction of this hypothetical model tree: IF *G*_1 _≤ 10 AND *G*_2 _> 10 THEN *TG *= 0.9 * *G*_2 _- 5. IF *G*_1 _> 10 THEN *TG *= 0.5 * *G*_1 _+ 1 The dot line is the results of apply the linear regression functions that estimate the target gene expression value.

Our approach takes each gene as a target gene and builds a model tree to predict the target gene expression values. By construction of model tree, linear regression functions are built to infer localized similarities over a more global similarity. Figure 3 presents a hypothetical example, the correlation between the target gene and two other genes is weak, however we can observe two strong local dependencies between them.

### Extracting gene-gene dependencies

This step extracts a set of dependencies between the target gene and the genes involved in the linear regression functions from each tree. Correlation-based methods extract gene-gene dependencies by computing a similarity score for each pair of genes. These methods are based on the assumption that two genes show similar expression profiles if they follow the same regulatory regime, i.e. coexpression hints at coregulation [11]. Our approach analyzes each gene as a target by taking into account the remaining genes as inputs to obtain linear models that estimate the expression value of that target gene. We assume that the genes involved in these linear models control or influence the target expression value and they follow the same regulatory regime. This influence can be explained when several genes fit a specific area of the space, which leads to an evidence for dependency.

Let *LM *be a multivariate linear model of a M5' tree defined by $LM:g_{x}=\sum_{i}\lambda_{i}g_{y_{i}}$, where *g*_*x *_belongs to the set of target genes, $g_{y_{i}}$, is a gene involved in the linear regression that belongs to the set of genes, and *λ*_*i *_is a coefficient of the linear model. Our approach considers that an hypothetical evidence of dependency or expression pattern exists between *g*_*x *_and every $g_{y_{i}}$, which will be statistically tested in the next step.

The output of this step is a set of gene-gene dependencies (*Q *in Figure 2) that are potential interactions for the problem under study.

### Building the gene regression network

After obtaining the set of gene-gene interactions, the significance of these results must be assessed. The authors in [32] have shown that for microarrays studies, the expected proportion of false discoveries among all the discoveries made (so-called *false discovery rate*, FDR) is more important than the low number of false discoveries or the small probability of making at least one false positive (calculated by means of adjustments of p-values). For this reason we apply a statistical procedure in order to control the number of type I errors (connections inferred which do not correspond to a connection in the real network, also called *false positives*) among the number of discoveries when a family of hypotheses is tested simultaneously.

Once the set of gene-gene dependencies (*D*) has been provided, our approach builds a graph *Q *of interactions defined as a tuple (*N, E*) of |*N*| nodes and |*E*| edges. We will denote by *g*_*x*_~*g*_*y *_an hypothetical gene-gene dependency. Our approach takes several *g*_*x*_~*g*_*y *_from *D *and the genes *g*_*x *_and *g*_*y *_are mapped as two nodes in the set of nodes *N*, and the dependency is mapped as an edge of the set *E*. This step, to decide which *g*_*x*_~*g*_*y *_is mapped onto an edge, i.e. which dependency is considered as a discovery, is carried out by means of the Benjamini-Yekutieli (BY) procedure.

The BY procedure is applied in order to test *m *null hypotheses $H_{0}^{1},H_{0}^{2},...,H_{0}^{m}$. Let *p*_1_,..., *p*_*m *_be the corresponding p-values to *m *null hypotheses. Let *p*_(1) _≤ *p*_(2) _≤ ... ≤ *p*_(__*m*__) _be the ordered p-values. This procedure defines *k *as detailed in Eq. 1 and rejects all hypothesis $H_{0}^{1},H_{0}^{2},...,H_{0}^{k}$.

(1) $$k=max\left\{i:p_{\left(i\right)}\frac{m}{i}\sum_{k=1}^{m}\frac{1}{k}\leq \alpha \right\}$$

If no such *i *exists, none of the hypotheses will be rejected. This procedure controls the proportion of false discoveries (FDR) among all the discoveries.

In this context, we will say that *g*_*x*_~*g*_*y *_is not an interaction in Q* if and only if there is not any significant monotonic relationship between the two variables, i.e. *H*_0 _: *ρ*_*xy *_≈ 0 (where *ρ *is a correlation measure), taking into account the subspace of the input data identified by the leaf of the linear model in the M5' tree. If this null hypothesis is rejected at the significance level represented by *α*, this dependency is mapped into the graph. To test whether a significant monotonic relationship exists, we use the Kendall's *τ *(under the subspace or subset of gene expression samples) as non-parametric measure of association [33].

### Algorithm

In order to formalize the algorithm, named REGNET, several definitions are required.

#### Definition 1 (Microarray)

*Let *M *be the microarray data, defined as *M=(C,G,ℒ), *where *$C=\{c_{1},c_{2},...,c_{n}\}$*is a finite set of experimental conditions*, $G=\{g_{1},g_{2},...,g_{m}\}$*is a finite set of genes, and *$ℒ=(v_{ij})$*is a n *× *m gene expression matrix, where v*_*ij *_= ℓ (*c*_*i*_, *g*_*j*_) *given by the level function *ℓ:C×G→ℝ.

#### Definition 2 (Partition)

*A partition *Π *of a set *S *is a non-empty collection of non-empty subsets of *S, Π = {*π*_*i*_}_*i *= 1__,..., *p *_*such that *⋃*π*_*i *_= S *and π*_*i *_⋂ *π*_*j *_= ∅ *when i *≠ *j for i, j *= 1,..., *p. The set of partitions of *S *is denoted by *PART(S).

#### Definition 3 (Model Tree)

*A model tree MT*_*j *_*is aimed at estimating the values of the level function ℓ for the column j, i.e. for the target gene g*_*j *_, *MT*_*j *_= {(*ψ*_*i*_, *ϕ*_*i*_)*}*_*i*__=1__,..., *q*_, *where *$\cup \psi_{i}\in \text{PART(}C\text{)}$, *and ϕ*_*i *_*is a linear function defined on a subset of genes *$\Omega_{i}\subset G-\{g_{j}\}$, *i.e., ϕ*_*i*_:Ω_*i *_→ ℝ. *Therefore, each function ϕ*_*i *_*will be applied in a subspace of conditions ψ*_*i *_*to locally estimate the level function of the gene g*_*j*_.

*Given a relative error threshold for the model tree θ, then *$MT_{j}^{\theta }$*defines a non-empty model tree when its relative error ε is smaller than θ*.

$$MT_{j}^{\theta }=\{\begin{array}{ll} MT & if\;\varepsilon <\theta \\ \emptyset & if\;\varepsilon \geq \theta \end{array}$$

#### Definition 4 (Forest)

*The forest of model trees FT is the collection of every model tree MT generated from each gene g*_*j*_, 1 ≤  *j *≤ *m, FT *= {*MT*_1_, *MT*_2_,..., *MT*_*m*_}, *where each MT*_*j *_*is built by minimizing the error ε at estimating the level function for gene g*_*j *_*and the conditions within ψ*_*i *_*by means of the functions ϕ*_*i*_.

#### Definition 5 (Association)

*A gene g is potentially associated with the gene g*_*j *_*(g *~ *g*_*j *_*) if g appears in any of the Ω*_*i *_*of the corresponding functions ϕ*_1_, *ϕ*_2_,..., *ϕ*_*q *_*defined at the leaves of the model tree MT*_*j *_, *whose target gene is g*_*j *_. *Each function ϕ*_*i *_*involves a set of genes *Ω_*i *_*related to g*_*j *_, *and therefore, all the genes associated with g*_*j *_, *represented as *$△(g_{j})=\cup_{{}^{i=1}}^{q}\Omega_{i}$, *constitute potential associations*.

*Given a threshold θ there is an association between two genes*, $g_{x}\overset{\theta }{\underset{}{\sim }}g_{y}$, *if and only if g*_*x *_*belongs to the set of genes that form the regression of g*_*y*_.

$$g_{x}\overset{\theta }{\underset{}{\sim }}g_{y}\Leftrightarrow g_{y}\in TG^{\theta }\wedge g_{x}\in \Delta (g_{y})$$

*where TG*^*θ *^*is the set of target genes*

$$TG^{\theta }=\{g_{j}\in G|MT_{j}^{\theta }\neq \emptyset \}$$

#### Definition 6 (Gene Regression Network)

*A gene regression network is a graph Q defined for a given θ as:*

$$Q=(TG^{\theta }\cup LG^{\theta },D^{\theta })$$

where LG is the set of associated genes

$$LG^{\theta }=\{g\in G|g\in \Delta (g_{i}),g_{j}\in TG^{\theta }$$

and D is the set of dependencies

$$D^{\theta }=\{(g_{x},g_{y})|g_{x}\overset{\theta }{\underset{}{\sim }}g_{y}\}$$

The input is the gene expression matrix *M*, a threshold value *θ *to prune the model trees generated, and the significance level *α *for the Benjamini-Yekutieli procedure. The output is a graph of interactions Q* among the genes in G.

Regarding the computational complexity of REGNET, the cost of building the forest of trees is *m *times the cost of building a M5' tree, i.e. *O*(*m*^2^*nlog*(*n*)), where *m *is the number of genes and *n *the experimental conditions; extracting the hypothetical dependencies is an iterative process which has a linear complexity *O*(*m*); and finally, the BY procedure involves sorting the p-values calculated before, i.e., *O*(*mlog*(*m*)). Consequently, the overall cost of the algorithm is *O*(*m*^2^*nlog*(*n*)).

## Results and Discussion

The robustness of the methodology is shown by means of the analysis on an *in silico *benchmark suite of datasets, the *Saccharomyces Cerevisiae *cell cycle and the *E. coli *data set.

### In silico benchmark suite of datasets

We tested our approach on a published in silico benchmark suite of datasets [34]. The goal is the prediction of network structure from the given in silico gene expression dataset. We use this suite as a blind performance test to compare our approach REGNET against several benchmark methods.

We used the simulated steady-state gene expression datasets reported in DREAM4 (In silico Network Challenge) [35]. The challenge is to infer 5 networks of size 100 hidden in 15 different experiments of microarray. For each network, the GNW tool [36] is used to simulate three different experiments of microarray: the steady-state levels of single-gene knockouts (deletions); knockdowns experiments by reducing the transcription rate of the corresponding gene by half; multifactorial experiment where each expression profile could be extracted from a patient.

For network inference, we applied several benchmark methods:

• A heuristic algorithm for learning high-dimensional dependency networks from genomic data. We used the *GeneNet *R package to infer causal networks based on partial correlations. *GeneNet *implements the methods of [37] and [38] for learning large-scale gene dependency networks.

• Weighted-LASSO for structured network inference implemented in the *Simone *R package [39] and [40]. This algorithm uses the GLasso procedure to estimate a sparse inverse covariance matrix using a lasso (L1) penalty.

• For learning Bayesian networks (BN) we used the R package named *Deal *[41] and the R package named *G1DBN *http://cran.r-project.org/web/packages/G1DBN.

Results reported here were obtained from *GeneNet*, *Simone *and *G1DBN*. The task of learning Bayesian Networks (BN) from data is NP-hard with respect to the number of network vertices, i.e. Bayesian methods are computationally intractable for a huge number of genes. The *Deal *algorithm for learning BN was unsuitable to obtained networks because of the number of genes in the input microarray (100 genes). The *G1DBN *was suitable to obtain networks because this algorithm performs Dynamic BN inference using first order conditional dependencies as heuristic.

**Figure 4:**
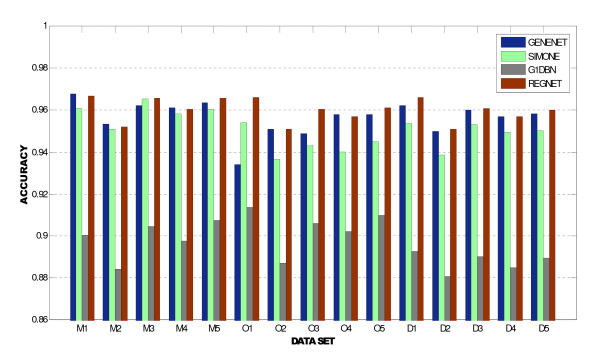
**Benchmark analysis**. Results reported by REGNET and the benchmark methods using the in silico benchmark suite of datasets [34]. The bars represent the accuracy of the prediction of network structure from the given in silico gene expression dataset.

Results reported by REGNET and the benchmark methods are shown in Figure 4. In this graphic, the accuracy is represented for each of the fifteen synthetic data sets. M, O and D represent the microarray data set obtained from a multifactorial, knockout and knockdown experiment, respectively. Results reported here by REGNET were obtained with *α *= 0.001.

Our approach outperformed the results reported by *G1DBN *and *SIMONE *in all the data set (knockout, knockdown and multifactorial experiments of microarray). In general, our approach showed higher accuracy. Only in five out of fifteen data sets, out approach did not outperform the results obtained by *GeneNet*.

### Saccharomyces Cerevisiae dataset

We use *Saccharomyces Cerevisiae *cell cycle expression data set [42], which contains 2884 genes and 17 experimental conditions. In the first experiment, the effect of pruning and non-pruning the forest of model trees is compared. Simplifying the forest involves rejecting all the M5' trees that have a relative error greater than a threshold. For both experiments a level *α *= 0.05 is fixed for the statistical BY procedure. To analyze the biological coherence of the results we use Gene Ontology attributes to characterize the resulted genes derived from our algorithm. We use FuncAssociate [43] to provide a measure (p-value) that determines whether the set of genes obtained is due to chance, or instead, to common biological behavior. Furthermore, this tool calculates appropriate corrections for multiple hypothesis testing, such as Westfall-Young [44].

**Figure 5:**
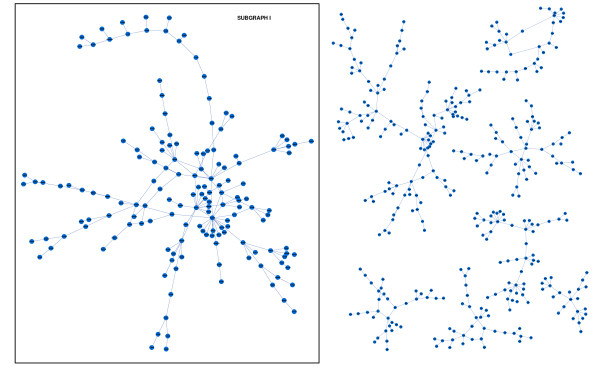
**Saccharomyces Cerevisiae dataset. Experiment I**. Gene regression network resulting from our approach at level *α *= 0.05 for the BY procedure. The image was created with Cytoscape [47]. This graph has 502 nodes and 504 edges. Subgraph I is the biggest subgraph obtained and it has 154 nodes and 162 edges.

Figure 5 depicts the experimental results, which consist of a network with eight main subgraphs or connected components. The algorithm also obtains other minor subgraphs (not depicted in the Figure) that are not considered because they are composed only by three or four edges. From these eight subgraphs, we calculated the correlation between pair of genes to obtain the number of weak correlated genes detected by our approach focused on localized similarities (see Additional file 1). We use the biggest subgraph in Figure 5, which has 154 genes, to analyze the result.

The resulted genes are functionally enriched for GO attributes and the great majority of these GO attributes are related with ribosome cellular component, as we can see in Table 1. This table reports these GO attributes, the number of genes in the subgraph with this attribute and the adjusted p-value less than *α *= 0.05 provided by the FuncAssociate tool [43]. In the first subgraph, there can be seen several genes related with the small subunit of the ribosome that is found in the cytosol (part of the cytoplasm that does not contain membranous or particulate subcellular components) of the cell. There are several genes that contribute to the structural integrity of these small ribosomal subunits which are involved in translation. Specifically, our approach has found genes related with the biological process of aggregation, arrangement and bonding together of constituent RNAs and proteins to form and maintain those small ribosomal subunits. In addition, there are several genes that are involved in the process of assembly and maintenance of the large subunit of the ribosome.

**Table 1:** Saccharomyces Cerevisiae data. Experiment I.

N	P-adj	GO Attribute
38	< 0.001	0005830: cytosolic ribosome

42	< 0.001	0005840: ribosome

37	< 0.001	0003735: structural constituent of ribosomal protein

46	< 0.001	0030529: ribonucleoprotein complex

20	< 0.001	0005843: cytosolic small ribosomal subunit

20	< 0.001	0015935: small ribosomal subunit

17	< 0.001	0015934: large ribosomal subunit

Gene Ontology attributes are used to characterize the genes obtained by our method from the yeast data set. It shows the biological analysis of the biggest subgraph I (see Figure 5). The first column represents the number of genes in the subgraph with this GO attribute, the second column is the adjusted p-value by Westfall and Young corrections and the third column is the name of the GO attribute.

**Figure 6:**
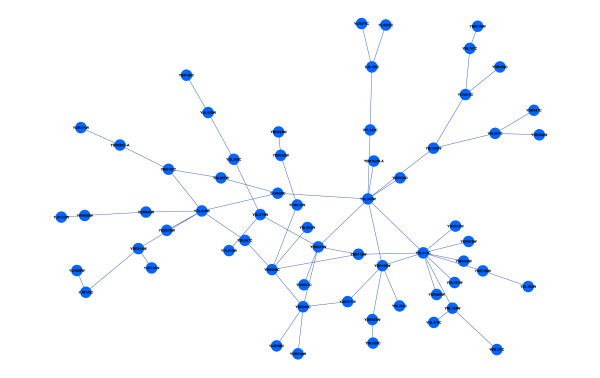
**Saccharomyces Cerevisiae dataset. Experiment II**. The biggest subgraph (62 genes) obtained from yeast Microarray data with a variation of our method, that consists in rejecting all the M5' that has a relative error greater than 50%.

We run our algorithm again but we introduce a variation that involves rejecting all the M5' that has a relative error greater than 50%. This variation restricts the number of linear models taken into account in the learning process of gene-gene interactions. Figure 6 shows the biggest subgraph obtained, which has 62 nodes and all of them belong to the first subgraph mentioned in Experiment I.

The main contribution of this variation is that the size of the subgraph is reduced more than 50% with respect to Experiment I, but the biological information is the same, as it can be noticed in Table 2. This table reports the biological study provided by GO database, that relates most of genes to ribosome cellular component (c.f. Table 1). In fact, all the GO attributes in Experiment I have remained in Experiment II, and they are obtained from the simplified forest (all the M5' trees have a relative error smaller than 50%).

**Table 2:** Saccharomyces Cerevisiae data. Experiment II.

N	p-adj	GO Attribute
21	< 0.001	0005830: cytosolic ribosome

23	< 0.001	0005840: ribosome

21	< 0.001	0003735: structural constituent of ribosomal protein

22	< 0.001	0005198: structural molecule activity

23	< 0.001	0030529: ribonucleoprotein complex

11	< 0.001	0005843: cytosolic small ribosomal subunit

11	< 0.001	0016283: eukaryotic 48S initiation complex

11	< 0.001	0016282: eukaryotic 43S preinitiation complex/eukaryotic 43S pre-initiation complex

25	< 0.001	0005829: cytosol

11	< 0.001	0015935: small ribosomal subunit

10	< 0.001	0005842: cytosolic large ribosomal subunit

24	< 0.001	0009059: macromolecule biosynthesis

23	< 0.001	0006412: protein biosynthesis

10	< 0.001	0015934: large ribosomal subunit

4	< 0.001	0000028: ribosomal small subunit assembly and maintenance

Biological analysis of the biggest subgraph from Experiment II (see Figure 6).

In summary, the use of constrains to provide more accurate model trees does not have negative influence on the quality of results. Selecting the best M5' trees from the forest reduces the size of the gene network without decreasing the quality of the results from a biological perspective.

### Escherichia coli dataset

The predictive performance of our approach was tested using Escherichia coli (E.coli) gene expression database from [45]. The E.coli gene expression database *M*^3^^*D *^(Many Microbe Microarrays Database) is used and *E_coli_v*3_*Build_*3 from T. Gardner Lab is built. This dataset consists of 524 arrays from 13 different collections corresponding to various conditions. The experiments were carried out on Affymetrix GeneChip E.coli Antisense Genome arrays, containing 4292 gene probes. A RMA normalization procedure was performed on the data prior to the application of our approach and the benchmark method.

Our approach REGNET and a gene relevance network method based on Partial Correlation were applied. Firstly, REGNET was applied several times with different values as a threshold of pruning phase: 25%, 50% and 100%. Second, the method proposed in [8] is used to provide partial Pearson and Spearman correlations (zeroth and first order correlations, with level *α *= 0.001, are calculated). Partial correlation coefficients quantify the correlation between two variables when conditioning on one or several other variables, which seems closer to causal relationships.

We chose the E.coli K12 transcriptional network in the Regulon database, version 6.3 [46] as true gene interaction network. From this transcriptional network we derived a gene association graph of 3288 interactions.

**Figure 7:**
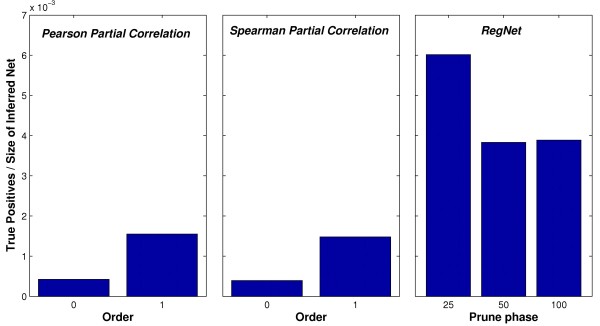
**Escherichia coli dataset**. Regulon Database is used to test the performance of REGNET and zeroth and first-order Pearson/Spearman correlation-based methods to build gene networks. The bars represent the proportion of true edges found in the gene network.

In absolute terms, there is a huge number of edges which does not correspond to any true edge from the Ecoli K12 transcriptional network. This situation shows the complexity of the gene expression regulation system. However, if we focus only on relative terms, i.e. the number of true positives divided by the size of the inferred network, we can observe that REGNET produces better results than the partial correlation-based methods. Figure 7 depicts the low proportion of true positives for each method. However, REGNET is much more selective, and builds smaller networks. For example, while 61 true edges are found in the REGNET network with 15908 interactions (0.0038), the smaller network obtained by a partial correlation-based method had 123 true edges in the network with 79372 interactions (0.0015), when using the first-order Pearson partial correlation. For zeroth-order partial correlations, the number of edges surpasses four millions of interactions.

## Conclusions

Inferring any type of relationship from data is a difficult task, particularly when non-linearity is present. Gene networks provide a framework to analyze regulation and causality.

Our approach, named REGNET, generates new hypothesis of interactions among genes from gene expression data, and differs from correlation-based methods in that the relationship between one gene and others is calculated simultaneously, and statistically validated when all these genes show linear dependency only in a region of the space. Our method is based on the idea that, given some control genes which define subspaces of the input data, multivariate linear models can be estimated for the target gene. REGNET strongly favours localized similarities over more global similarity, which it is one of the major drawbacks of correlation-based methods.

Experimental results show the good performance of REGNET. The first experiment, with yeast cell cycle data, is consistent with Gene Ontology. The aim of the second experiment is to check the ability of finding true gene associations from gene expression data in comparison with E.coli transcriptional network from Regulon database.

In general, REGNET is a powerful method to hypothesize on unknown relationships, and therefore, on genes potentially related to biological functions.

## Authors' contributions

IN refined the method and designed the experiments for testing the performance of REGNET. JAR conceived the method and leaded the project. JRS critically revised the computational and statistical steps of the method. All authors read, edited and approved the final manuscript.

## Supplementary Material

Additional file 1 **yeastSubNET1-8.xls**. Gene-gene associations resulting from our approach using *Saccharomyces Cerevisiae *data as input. The correlation measure between pair of genes from the network is reported, together with the number of weak correlated genes detected by our approach focus on localized similarities. Click here for file
